# Effect of Microgravity on the Gut Microbiota Bacterial Composition in a Hindlimb Unloading Model

**DOI:** 10.3390/life12111865

**Published:** 2022-11-12

**Authors:** Ruqaiyyah Siddiqui, Rizwan Qaisar, Naveed Ahmed Khan, Ahmad M. Alharbi, Hasan Alfahemi, Adel Elmoselhi

**Affiliations:** 1 College of Arts and Sciences, American University of Sharjah, Sharjah 26666, United Arab Emirates; 2 Department of Medical Biology, Faculty of Medicine, Istinye University, Istanbul 34010, Turkey; 3 Department of Basic Medical Sciences, College of Medicine, University of Sharjah, Sharjah 27272, United Arab Emirates; 4 Department of Clinical Sciences, College of Medicine, University of Sharjah, Sharjah 27272, United Arab Emirates; 5 Department of Clinical Laboratory Sciences, College of Applied Medical Sciences, Taif University, Taif 21944, Saudi Arabia; 6 Department of Medical Microbiology, Faculty of Medicine, Al-Baha University, Al-Baha 65799, Saudi Arabia

**Keywords:** gut microbiome, microgravity, rodent hindlimb unloading model, metabolites

## Abstract

We utilised a ground-based microgravity hindlimb unloading (HU) mouse model to elucidate the gut microbiota bacterial changes in mice under a simulated microgravity environment. Four-month-old, male C57/Bl6 mice were randomly divided into ground-based controls and the HU groups and kept under controlled environmental conditions. For the microgravity environment, the mice were suspended in special cages individually for 20 days. At the end of the suspension, the mice were sacrificed; gut dissections were performed, followed by a metagenomic analysis of bacterial species, which was carried out by extracting DNA and 16S rRNA analysis. The results revealed that the gut bacterial communities of mice under gravity and microgravity were different. Notably, our findings revealed differences in the bacterial community structure. Around 449 bacterial OTUs were specific to mice kept under normal gravity versus 443 bacterial OTUs under microgravity conditions. In contrast, 694 bacterial OTUs were common to both groups. When the relative abundance of taxa was analyzed, Bacteroidetes dominated the gut (64.7%) of normal mice. Conversely, mice in the microgravity environment were dominated by Firmicutes (42.7%), and the relative abundance of Bacteroidetes differed significantly between the two groups (*p* < 0.05). The distribution of Muribaculaceae between normal mice versus microgravity mice was significantly different, at 62% and 36.4%, respectively (*p* < 0.05). Furthermore, a significant decrease in 11 bacteria was observed in mice under simulated microgravity, including *Akkermansia muciniphila*, *Eubacterium coprostanoligenes*, *Bacteroides acidifaciens*, *Clostridium leptum*, *Methylorubrum extorquens*, *Comamonas testosterone*, *Desulfovibrio fairfieldensis*, *Bacteroides coprocola*, *Aerococcus urinaeequi*, *Helicobacter hepaticus*, and Burkholderiales. Further studies are needed to elucidate gut bacterial metabolites of these identified bacterial species in microgravity conditions and normal environment. Notably, the influence of these metabolites on obesity, neuroprotection, musculoskeletal and cardiovascular dysfunction, longevity, inflammation, health, and disease in astronauts ought to be investigated and will be important in developing procedures against adverse effects in astronauts following space travel.

## 1. Introduction

Astronauts have been traveling to space since 1961, and factors such as microgravity and radiation exposure are known to cause physiological changes [1]. Nonetheless, the role of the gut microbiome and its impact on astronaut health is incompletely understood [2,3]. Previous work has mainly focused on other facets of astronaut health, for instance, visual impairment and the cardiovascular and musculoskeletal system, but the detailed impact of microgravity environments experienced during spaceflight on the gut microbiome and its effect on host health and how these can be reversed and/or prevented remains to be elucidated. A recent study by the National Aeronautics and Space Administration (NASA) compared the changes in the gut microbiome of an astronaut and his twin brother (who was based on Earth during the mission), termed the “NASA twins study” [4]. The study depicted a modification in the gut microbiome between the twins, and numerous changes in the microbiome composition and diversity were observed in the astronaut, but not seen in his twin brother on Earth [4]. These are intriguing findings; however, testing of interventional and/or preventative strategies remains untested in humans. Previous studies indicate that intestinal homeostasis disorders, for instance, decrease in absorption and digestion, intestinal structure disruption, immunity dysfunction, and dysbiosis of the microbiome, can be induced by simulated weightlessness and microgravity [5,6,7]. Thus, comprehending the precise changes in the gut microbiome is warranted, given the profound influence of the microbiome on its host and overall impact on human health.

A substantial study designated “the Astronaut Microbiome Project” is being accomplished to elucidate the astronaut microbiome with the aim to determine microbiome changes during space travel [8]. This study has depicted an increase in *Faecalibacterium*, a beneficial butyrate producer. Conversely, species associated with chronic inflammation of the intestine, namely *Parasutterella* were observed. Furthermore, a decrease in *Akkermansia*, that is associated with anti-inflammatory properties, were seen. Therefore, prebiotics containing *Akkermansia*, to diminish diseases concomitant with inflammatory responses, have been suggested [8,9].

Herein, we utilised a hindlimb unloading (HU) mouse model, which has been well ascertained as a ground-based microgravity model. The model involves suspending the mouse from its tail in a cage, so that the hindlimbs are elevated from the ground with a 30-degree head-down tilt. Due to mechanical unloading of the hindlimbs and cephalic fluid shifts, the mouse partly recapitulates the simulated microgravity conditions similar to astronauts in space. Several physiological changes are reported in these mice, including blood redistribution, cephalic fluid shift, and insufficient oxygen and blood supply to the gastrointestinal tract [10,11]. We conducted gut metagenomic DNA extractions in both the hindlimb suspension mouse model, mimicking a microgravity environment, and mice under normal gravity conditions to elucidate the gut microbiome composition in those conditions. These findings will help consolidate previous studies and further elucidate the precise gut microbiome differences under microgravity conditions versus normal gravity environment in vivo, with the long-term aim to design and test, pre-, and post-biotics to modulate astronauts’ health.

## 2. Materials and Methods

### 2.1. Animals

Protocols for animals were sanctioned and approved by University ACUC (Animal Care and Use Committee) in agreement with accepted international standards.

### 2.2. The HU Mice Model

For the HU model, 4-month-old male c57BL/6j mice were utilized. This age is considered a mature adult age for mice, which is equivalent to approximately 30-year-old humans [12]. This age is corresponding to nearly the average age of astronauts in flight and encompasses an adequate proportion of human patients on bed rest with prolonged inactivity [13,14]. The mice were randomly divided into ground-based controls and the HU groups. Mice were kept under controlled environmental conditions (20 ± 1 °C, with light/dark periods of 12 h each) with food (standard chow diet for mice) and water provided ad-libitum. The mice of the HU group were suspended, individually, in specially designed cages by a thin string tied at one end to the tail and at the other end to the top of the cage (one mouse/cage) [15]. The experimental group (n = 3 mice) was suspended for 20 days (the duration was chosen based on our previous experiments because it shows changes in the organs and body weights following HU) [16]. At the end of the suspension, the mice were released from the string and immediately euthanized via cervical dislocation.

### 2.3. Gut Dissection and Sample Collection

After euthanasia, the stomach and small and large intestines of control and HU mice were dissected immediately, frozen in liquid nitrogen, and deposited at −80 °C for further molecular studies.

### 2.4. Metagenomic DNA Extraction

The metagenomic studies were performed by extracting DNA from the mice gut as described before [17]. Briefly, each sample was incubated with 1 mL of pre-heated lysis buffer (20 g sodium dodecyl sulfate per litre, 0.1 M Tris-HCl, 0.15 M sodium chloride, 25 mM EDTA) and pH adjusted to 8.0. Next, 10 μL of Proteinase K (10 mg per mL) was added, and samples were kept at 65 °C for 60 min. Following centrifugation at 12,000× *g* for 10 min, the supernatant was first extracted with phenol/chloroform/isoamyl alcohol at a ratio of 25:24:1 (*v*/*v*/*v*) and then with chloroform/isoamyl alcohol at a ratio of 24:1 (*v*/*v*). Next, DNA was pelleted using potassium acetate (3 M, pH 5.5) and 95% ethanol, followed by centrifugation at 15,000× *g* for 10 min. The pellet was washed twice with 70% ethanol and then dissolved in 0.4 mL of buffer (10 mM Tris, 1 mM EDTA). Next, samples were incubated with 10 mg of RNase for 30 min at 37 °C to remove residual RNA. This was followed by extraction with chloroform/isoamyl alcohol to remove protein. Finally, the upper layer was collected in a tube containing 2.5 vol of ethanol (second precipitation) to precipitate the DNA and centrifuged for 10 min at 15,000× *g*. The DNA pellet was washed twice, dried, and then re-suspended in 0.2 mL dH_2_O. DNA purity and concentration were elucidated using 1% agarose gels [18,19]. Accordingly, the DNA was diluted to 1 ng per μL with sterile water.

### 2.5. Sequencing of the Bacterial 16 rRNA

The 16S rRNA/18S rRNA/ITS genes in distinct regions (V3-V4/16S) were amplified with specific primers (341F-CCTAYGGGRBGCASCAG, and 806R-GGACTACNNGGGTATCTAAT). All PCR mixtures contained 15 μL of Phusion^®^ High-Fidelity PCR Master Mix (New England Biolabs, Ipswich, MA, USA), 0.2 μM of each primer and 10 ng target DNA, and cycling conditions consisted of a first denaturation step at 98 °C for 1 min, followed by 30 cycles at 98 °C (10 s), 50 °C (30 s) and 72 °C (30 s), and a final 5 min extension at 72 °C. An equal volume of 1X loading buffer (containing SYB green) was mixed with PCR products and electrophoresed on 2% agarose gel [20]. The PCR products (470 bp) were mixed in equal proportions, and then Qiagen Gel Extraction Kit (Qiagen, Hilden, Germany) was used to purify the mixed PCR products. Sequencing libraries were generated with NEBNext® Ultra™ IIDNA Library Prep Kit (Cat No. E7645). The library quality was evaluated on the Qubit@ 2.0 Fluorometer (Thermo Scientific, Waltham, MA, USA) and Agilent Bioanalyzer 2100 system. Finally, the library was sequenced on an Illumina NovaSeq platform and 250 bp paired-end reads were generated. Quality filtering on raw tags was accomplished through specific filtering conditions in order to obtain high-quality clean tags, according to the Qiime 2 [21] for subsequent analysis.

### 2.6. Data Analysis

As sequencing resulted in some “dirty data”, the raw data was merged and filtered to obtain clean data to make the information analysis accurate and reliable. Briefly, paired-end reads were assigned to samples based on their unique barcodes and were truncated by cutting off the barcodes and primer sequences. Paired-end reads were merged using FLASH (Version 1.2.11, http://ccb.jhu.edu/software/FLASH/, accessed on 1 March 2022), a very fast and accurate analysis tool designed to merge paired-end reads when at least some of the reads overlap with the reads generated from the opposite end of the same DNA fragment, and the splicing sequences were called Raw Tags. Quality filtering on the raw tags was performed using the fastp (Version 0.20.0) software to obtain high-quality Clean Tags. The Clean Tags were compared with the reference database (Silva database https://www.arbsilva.de/for 16S/18S, accessed on 2 March 2022, and Unite database https://unite.ut.ee/for ITS, accessed on 2 March 2022) using Vsearch (Version 2.15.0) to detect the chimera sequences, and then the chimera sequences were removed to obtain the Effective Tags. For the Effective Tags obtained previously, denoise was performed with DADA2 or deblur module in the QIIME2 software (Version QIIME2-202006) to obtain sequence variants, and then variants with abundance less than 5 were filtered out. Species annotation was performed using QIIME2 software. For 16S/18S, the annotation database is Silva Database, while for ITS, it is Unite Database. In order to study the phylogenetic relationship and the differences of the dominant species among different samples (groups), multiple sequence alignment was performed using QIIME2 software. The absolute abundance of variants was normalized using a standard sequence number corresponding to the sample with the least sequences. Subsequent analysis of alpha diversity and beta diversity were all performed based on the output of normalized data. In order to analyze the diversity, richness and uniformity of the communities in the sample, alpha diversity was calculated from 7 indices in QIIME2. In order to evaluate the complexity of the community composition and compare the differences between samples (groups), beta diversity was calculated in QIIME2. To study the significance of the differences in community structure between groups, the adonis and anosim functions in the QIIME2 software were used to do analysis. To find out the significantly different species at each taxonomic level (Phylum, Class, Order, Family, Genus, Species), the R software (Version 3.5.3) was used to do MetaStat and *t*-test analysis.

## 3. Results

### 3.1. Characteristics of Study Subjects

At the start of the experiment, there was no significant difference in the body weights of control and HU mice (controls; 27.3 ± 2.4, HU; 26.9 ± 2.3, *p* = 0.231). HU for 20 days resulted in a significant reduction in body weights (controls: 27.8 ± 2.5, HU: 25.2 ± 2.1, *p* < 0.05). The HU mice also showed alterations in the weights of other body organs, including atrophy of hindlimb muscles, thus validating the mouse model. On the other hand, we did not observe any significant reduction in mice behavior, physical activity, or food and water intake.

### 3.2. Composition of Microbial Community Analysis between Gravity and Microgravity Groups: Interspecific Variations in Bacterial Gut Communities

Based on high-quality sequences, two groups were obtained. Next, 2479 valid tags with better quality remained (1250 and 1229 tags for Group A and Group B, respectively). Next, OTU classification was accomplished on all high-quality sequences with 97% similarity, utilising the Uparse software, and sequences with the largest abundance within OTUs were selected as representative sequences to be analysed in the Silva-16S database. The data depicted a total of 3898 OTUs, that is, 2015 and 1883 OTU for Group A and Group B, respectively.

**Figure 1:**
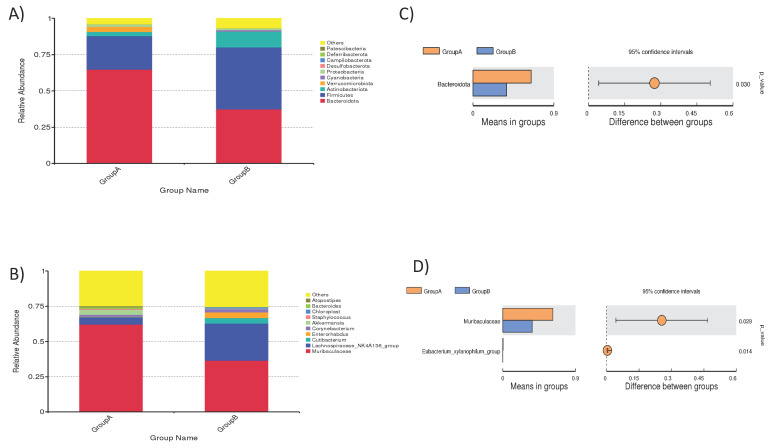
(**A**) Distribution of dominant bacterial communities in mice under a gravity environment (labelled as Group A), and (**B**) under a microgravity environment (labelled as Group B), respectively. (**C**,**D**) show examples of significantly different (*p* < 0.05) phyla and genera, respectively.

The gut bacterial communities of gravity and microgravity were different, as shown in Figure 1. Around 449 bacterial OTUs were specific to the gut of mice kept under normal gravity (Group A), while 443 bacterial OTUs were specific to the gut of mice kept under microgravity conditions (Group B). In contrast, 694 bacterial OTUs were common to both Groups. A list of all bacterial OTUs observed in both groups is shown in the Supplementary File (Supplementary Table S1). Furthermore, Anosim analysis (nonparametric test) was used to evaluate whether the variation among groups is significantly larger. The results revealed a positive R value of 0.8889.

### 3.3. Relative Abundance of Taxa

Taxonomic annotation data revealed the top 10 taxa of each group at a different taxonomic rank (phylum, class, order, family, genus), and these were designated to show distribution histograms of relative abundance of taxa to visualize taxa with higher relative abundance and proportion in different classification levels of each sample. The relative abundance of taxa in the phylum and genus is shown in Figure 1. “Others” represents the total relative abundance of the rest of the phyla besides the top 10 (Supplementary Table S2). Interestingly among phyla, Bacteroidota represented the highest abundance (64.7%) in normal mice. In contrast, Firmicutes represented the highest abundance (42.7%) in mice under a microgravity environment. The relative abundance of Bacteroidota differed significantly between the two groups (Figure 1). Overall, the relative abundance of the top 10 phyla in normal mice was in the order of Bacteroidota, Firmicutes, Actinobacteriota, Verrucomicrobiota, Cyanobacteria, Proteobacteria, Desulfobacterota, Campilobacterota, Deferribacterota, and Patescibacteria. In contrast, the relative abundance of top 10 phyla in mice kept under a microgravity environment was in the order of Firmicutes, Bacteroidota, Actinobacteriota, Cyanobacteria, Proteobacteria, Desulfobacterota, Verrucomicrobiota, Campilobacterota, Deferribacterota, and Patescibacteria. The distribution of Muribaculaceae between normal mice versus microgravity mice was significantly different, at 62% and 36.4%, respectively (*p* < 0.05) (Figure 1D). Likewise, differences were observed between other genera in both groups (the top 10 taxa based on their relative abundance were selected) (Figure 1). Overall, the relative abundance of the top 10 in normal mice were observed in the order of Muribaculaceae, Lachnospiraceae, *Akkermansia*, *Corynebacterium*, *Bacteroides*, *Atopostipes*, *Staphylococcus*, *Cutibacterium*, *Enterorhabdus*, and *Chloroplast*. In contrast, the relative abundance of top 10 in mice kept under a microgravity environment were observed in the order of Muribaculaceae, Lachnospiraceae, *Enterorhabdus*, *Cutibacterium*, *Corynebacterium*, *Chloroplast*, *Staphylococcus*, *Akkermansia*, *Bacteroides*, and *Atopostipes* (Figure 1B).

**Figure 2:**
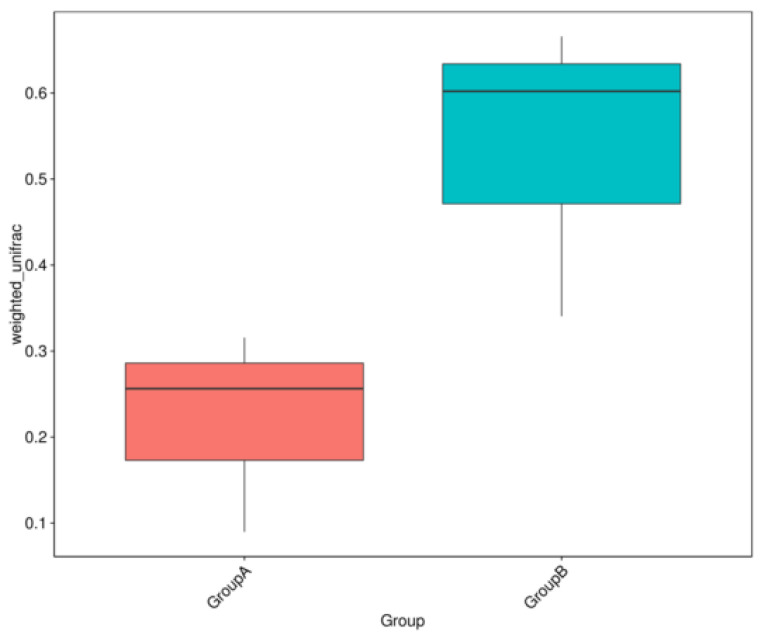
Boxplots based on Weighted Unifrac distance between mice under a gravity environment (Group A) and mice under a microgravity environment (Group B), respectively.

A boxplot was generated to show the difference of Beta Diversity indices between groups. The data indicate that both groups were different in bacterial community structure (Figure 2).

Finally, MetaStats analysis was performed to determine differences between the two groups. The results depicted that 11 species were significantly different between the two groups including, *Akkermansia muciniphila*, *Eubacterium coprostanoligenes*, *Bacteroides acidifaciens*, *Clostridium leptum*, *Methylorubrum extorquens*, *Comamonas testosterone*, *Desulfovibrio fairfieldensis*, *Bacteroides coprocola, Aerococcus urinaeequi*, *Helicobacter hepaticus*, and order Burkholderiales (Table 1).

## 4. Discussion

The gut microbiome is comprised of circa 100 trillion microorganisms (predominantly bacteria, but also fungi, protozoa, and viruses) and encodes more than 3 million genes that produce thousands of metabolites, with many functions that impact the overall health of the host [22,23]. Various reports indicate that the microbiome can impart protection against many disorders, such as systemic metabolic disease (type 2 diabetes and obesity), inflammatory bowel disease, eczema, and allergic diseases, whereas dysbiosis in the gut, can in turn, lead to the development of disease as well as affect the immune system [24,25,26]. The role of the gut microbiome in astronaut health is only recently becoming apparent, as depicted in the NASA twins study which revealed decreases in bacterial diversity and the overall composition of the gut microbiome was affected, but the precise effects on astronaut health are unknown [27]. Moreover, the study of the gut microbiome in astronauts has limitations. For instance, the NASA twins study only analysed a single astronaut. Further studies with more astronauts are needed to substantiate and corroborate these findings. Although other studies have been accomplished, such as analogous missions based on earth, namely the “MARS500 study”, wherein the microbiome of astronauts were analysed over 520 days [9], in vivo studies in simulated microgravity are of importance to identify changes in microbial diversity and composition and to subsequently test pre- and/or post-biotics. It is anticipated that our understanding of microbiome modulation under a microgravity environment would help in the rationale design of preventative strategies.

In the present study, we employed the hindlimb suspension mouse model, a ground-based in vivo microgravity model that mimics physiological changes concomitant with space travel [28,29]. A limitation in our study was that we used only 4-month-old male mature adult mice; thus, investigating older and female mice for the same model is definitely warranted since the age may alter the gut microbiota [30,31]. In our study, we accomplished gut metagenomic DNA extractions in both the hindlimb suspension mouse model and in mice under normal conditions and elucidated the gut microbiome composition. It is well known that two taxa of bacteria inhabit the human gut, specifically the Bacteroidetes and the Firmicutes [32]. Despite the limitation of the small sample size in the current study, we found statistically significant differences among the two groups of mice, which strengthens our confidence in the biological relevance of our data, similar to a recent report from our laboratory [16]. The results of our study revealed that the relative abundance of Bacteroidetes and Firmicutes was different between the two groups. These results are in agreement with previous studies, whereby it was revealed that the abundance of 17 gastrointestinal genera was altered during space travel [8].

Of note, the data from our study depicted reduced levels of *Akkermansia* *muciniphila* in mice in a simulated microgravity environment in comparison to normal mice, which is interesting as this genus is typically correlated with anti-inflammatory properties [33]. This data corroborated the data from the “astronaut microbiome project”, which also depicted a decrease in *Akkermansia*, and, therefore, pre-biotics comprising *Akkermansia* to reduce the chance of diseases associated with chronic inflammatory responses has been proposed [8,9].

Our data also depicted a decrease in *Eubacterium coprostanoligenes* in mice under simulated microgravity in comparison to normal mice. It is recognised that members of the genus *Eubacterium* spp. produce butyrate and may play a pivotal role in the suppression of inflammation and immunomodulation of the gut [34]. *Eubacterium* spp. also participates in bile acid and cholesterol transformations and contributes to gut homeostasis. Gut dysbiosis and modifications in *Eubacterium* spp. within the gut have been associated with disease states [34]. Furthermore, *Eubacterium* spp. has been depicted to carry out metabolic alterations in the gut with beneficial effects on human health, such as the detoxification of toxic compounds into non-toxic states [34]. Our data also indicated a noteworthy decrease in the relative abundance of Burkholderiales in mice under simulated microgravity compared to normal mice. Previously, it was reported that *Oxalibacterium formigenes*, a betaproteobacterium within the order Burkholderiales, is one of the few bacteria in the colon with well described health benefits and regulates the homeostasis of oxalic acid while also preventing kidney stone formation [35].

Firmicutes and Bacteroidetes are the two most important bacterial phyla that inhabit the gut and play a pivotal role in maintaining homeostasis in the host [36]. A balanced proportion of these phyla is imperative for maintaining overall health. Modifications in these proportions are associated with dysbiosis of the gut and can lead to obesity and inflammatory bowel disease [36]. Firmicutes are gram-positive and play a fundamental function in the metabolism and nutrition of the host through short-chain fatty acid synthesis and are involved in the regulation of satiety and hunger. Conversely, Bacteroidetes are gram-negative and associated with immunomodulation and their constituents interact with cell receptors and augment immune reactions through synthesis of cytokines [36].

## 5. Conclusions

In conclusion, the data from our study depicted the translational value of the in vivo hindlimb suspension microgravity mouse model, and the gut microbiome composition in normal conditions as well as simulated microgravity conditions were determined. This is important as in vivo studies can help identify changes in microbial diversity and composition and to subsequently test pre- and/or post-biotics. Based on our findings, several bacterial species were identified that depicted a significant decrease in microgravity conditions compared to normal mice and alterations in the ratio of Bacteroidetes to Firmicutes was observed under simulated microgravity conditions. Bacterial species that were significantly decreased should be isolated in future studies, and condition media containing their metabolites should be prepared and investigated in order to prepare pre/probiotics. These should then be tested using this model in microgravity and normal conditions. Furthermore, given the logistical issues of testing directly in astronauts, species already identified in the “astronaut microbiome project”: such as *Akkermansia*, should also be tested in vivo in mice as pre/probiotics. Future studies are needed to elucidate metabolites produced by gut bacteria in microgravity conditions in contrast to normal environments. Notably, the influence of these molecules on obesity, neuroprotection, longevity, inflammation, health, and disease in astronauts needs to be clarified. These studies will be important in developing appropriate procedures against the adverse modifications observed in astronauts following space travel.

## Figures and Tables

**Table 1:** MetaStats analysis with significant differences between representative bacterial communities in mice under gravity environment (Group A) and under microgravity environment (labelled as Group B), respectively. *t*-test is performed to determine bacteria with significant variation between groups (*p* value < 0.05) at various taxon ranks including phylum, class, order, family, genus, and species.

Species	Mean	Variance	SE	Mean	Variance	SE	*p* Value
*Akkermansia muciniphila*	0.03397	0.000305	0.010088	0.000896	5.32 × 10^−7^	0.000421	0.004909
*Eubacterium coprostanoligenes*	0.00446	5.00 × 10^−6^	0.00129	0.000354	2.27 × 10^−9^	2.75 × 10^−5^	0.006939
*Bacteroides acidifaciens*	0.00083	1.17 × 10^−7^	0.000197	3.79 × 10^−5^	4.30 × 10^−9^	3.79 × 10^−5^	0.00303
*Clostridium leptum*	0.00055	1.42 × 10^−7^	0.000218	4.42 × 10^−5^	2.27 × 10^−9^	2.75 × 10^−5^	0.014111
*Methylorubrum extorquens*	0.00018	2.38 × 10^−8^	8.91 × 10^−5^	0	0	0	0.034232
*Comamonas testosterone*	8.84 × 10^−5^	5.14 × 10^−9^	4.14 × 10^−5^	0	0	0	0.018808
*Desulfovibrio fairfieldensis*	0.0001	8.01 × 10^−9^	5.17 × 10^−5^	0	0	0	0.046788
*Bacteroides coprocola*	8.21 × 10^−5^	5.14 × 10^−9^	4.14 × 10^−5^	0	0	0	0.03804
*Aerococcus urinaeequi*	8.84 × 10^−5^	5.86 × 10^−9^	4.42 × 10^−5^	0	0	0	0.022596
*Helicobacter hepaticus*	5.68 × 10^−5^	2.51 × 10^−9^	2.89 × 10^−5^	0	0	0	0.041848
Burkholderiales	2.53 × 10^−5^	4.78 × 10^−10^	1.26 × 10^−5^	0	0	0	0.022596

## Data Availability

The data are available at: https://doi.org/10.34740/KAGGLE/DSV/4403892.

## Supplementary Materials

The following supporting information can be downloaded at: https://www.mdpi.com/article/10.3390/life12111865/s1, Table S1: A list of all bacterial OTUs observed in both groups. Table S2: Significant differences and the correspondent *p* values.
